# From temperament to YouTube: exploring the link between childhood temperament, YouTube usage patterns, and emotional/behavioral problems among children

**DOI:** 10.1186/s12889-024-19011-w

**Published:** 2024-06-07

**Authors:** Donghee Kim, Sangha Lee, Hyojin Kim, Yunmi Shin

**Affiliations:** 1 Department of Psychiatry, Ajou University School of Medicine, Suwon, Republic of Korea; 2 Department of Medical Sciences, Graduate School of Ajou University, Suwon, Republic of Korea

**Keywords:** YouTube, Temperament, Usage, Emotional problems, Behavioral problems, Children

## Abstract

**Background:**

YouTube is a widely used video sharing and social networking platform among children and adolescents. However, research on YouTube usage among this population remains scarce. Specifically, studies on factors that influence children and adolescents' usage are clinically significant but largely lacking. Additionally, few studies have examined the association between usage and emotional/behavioral problems, which is fundamental to smartphone research. Therefore, this study explored the relationship between early childhood temperament, subsequent YouTube usage patterns, and emotional/behavioral problems.

**Methods:**

The Kids Cohort for Understanding Internet Addiction Risk Factors in Early Childhood (K-CURE) is the first long-term prospective cohort study in Korea aimed at understanding the long-term effects of media exposure on young children. The study included 195 children aged 8–11 years enrolled in the K-CURE study. Caregivers, predominantly mothers, who voluntarily participated during their visits to community centers for children’s mental health in Korea’s major cities, completed a detailed self-administered survey. Childhood temperament was measured in 2018 when the children were 5–8 years old. Subsequent YouTube usage patterns and emotional/behavioral problems were assessed in 2021. Data were analyzed using frequency analysis, correlation analysis, and multiple linear regression.

**Results:**

The study found that 21.0% of children started using YouTube before age 4, with the most common onset age being 8–9 years (30.3%). These children used YouTube on average 4.8 days per week for 68.5 min per day. Early childhood persistence was negatively associated with the subsequent YouTube usage duration, and the age at first YouTube use was negatively correlated with subsequent usage frequency. Furthermore, a younger age at first YouTube use and higher usage frequency were significantly associated with increased emotional/behavioral problems.

**Conclusions:**

In the YouTube environment, where content is automatically recommended based on user preferences, traits related to usage patterns may be associated with persistence, which is linked to self-regulation. Considering the current trend where children use smartphones, contents frequently for very short durations, our findings highlight the importance of self-regulation in the media usage of children who are still developing. Additionally, our results provide fundamental information for future YouTube studies and illustrate similarities and differences between smartphone and YouTube research.

## Background

The use of mobile devices has continued to grow with the advancement of technology. In 2018, the smartphone ownership rate among elementary school students in South Korea was 62.9%. This percentage increased to 93.9% in 2022, implying that 9 out of 10 elementary school students owned a smartphone [1]. Children and adolescents use smartphones mainly for watching videos, social networking, gaming, and browsing the internet. The younger the user, the more likely they are to watch broadcasts and videos. Notably, YouTube is the most widely used application among children to watch videos [1, 2].

YouTube is a popular video-sharing platform freely available on television, computers, and smartphones [3]. It has localized versions for more than 100 countries and is available in 80 languages. Initially, its main purpose was to share videos, but, today, it serves many purposes, such as providing knowledge, entertaining viewers, and facilitating social networking. More than 2 billion people log onto YouTube monthly, and, on average, it is used for more than 1 billion hours daily [4]. In 2015, YouTube also launched YouTube Kids to provide more kid-friendly video content, further reaching out to younger viewers.

The growing popularity of YouTube has drawn attention in relation to the already prominent issues surrounding internet and smartphone usage. Previous research on internet and smartphone usage has consistently shown that excessive use of these technologies is associated with high levels of emotional difficulties, problematic behaviors, and lower levels of adjustment. It has been observed that children and adolescents who used smartphones more are more likely to exhibit internalizing symptoms such as depression and loneliness, as well as externalizing symptoms like aggressive behaviors in the future [5, 6].

YouTube is also an internet, smartphone-based medium, so its findings may be similar to those of previous internet, smartphone studies. A recent study found that higher levels of YouTube addiction were also associated with higher levels of internalizing and externalizing problem behaviors [7]. Along with smartphones, social media also has been studied extensively due to the increasing use of social network services, but each social media platform, such as Facebook, Instagram, Twitter and YouTube, has its own unique structure, norms, and culture [8]. While Facebook is a service that allows users to connect and interact through posts based on shared interests, Instagram is a social network that fosters connections by sharing daily life through photos and videos, rather than text-based posts. YouTube, however, distinguishes itself by focusing on video content and fosters a unique culture of building relationships among anonymous users through interactive features such as Likes, Comments, and Followers [9, 10]. Overall, YouTube can be considered a platform that fuses the traditional media elements of television, movies, and music with the interactive elements of digital media. Since each platform, utilized through smartphones as a tool, has its own distinct nature, it cannot be assumed that research findings concerning YouTube will necessarily align with those of existing studies on smartphones and other social media platforms.

However, research on YouTube is still relatively scarce compared to the extensive studies conducted on internet and smartphone usage. Particularly, the use of YouTube, can have more detrimental effects on children and adolescents who are in their developmental stages [11–13]. In this context, research into the factors influencing YouTube usage patterns is essential, yet there has been little focus on this aspect concerning YouTube. As the use of YouTube by children and adolescents is rapidly increasing worldwide, it is critically important to explore the factors that influence their usage patterns.

Previous studies on smartphones or social media have identified several risk factors for excessive or problematic smartphone use, including sociodemographic and personality factors, psychological conditions, purpose of use, and usage patterns [14]. Recent research on YouTube has examined the relationship between child YouTube usage and various factors: dispositional factors such as anxiety, developmental factors such as inattention and self-regulation, and social factors such as parental and peer YouTube usage. The study found significant associations between anxiety, inhibitory control, and child YouTube usage [15]. In addition to anxiety, which was examined in this study, temperament is another stable and important dispositional factor that should be considered. Temperament refers to the manner and intensity of one’s reaction to external stimuli and one’s ability to regulate and control oneself [16]. Investigating the relationship between temperament as dispositional factor and subsequent YouTube usage patterns among children and adolescents would be meaningful. However due to the challenges of conducting such research and the fact that studies on YouTube have only recently become prevalent, no research currently exists addressing this specific association.

Furthermore, there is very little research available on YouTube concerning fundamental topics in smartphone usage studies, such usage including as the age of first use, usage duration, and usage frequency, and their associations with emotional and behavioral problems in children and adolescents. Existing studies on YouTube for children and adolescents have mainly focused on content analysis to evaluate the effectiveness, functionality, and appropriateness of content [17, 18]. They have also investigated the effects of advertisements and inappropriate videos on this age group [19].

Research on smartphone and social media use is also largely focused on late adolescents and young adults due to the high rates of smartphone ownership and use among this age group [14, 20]. However, recent data show that early children are beginning to use YouTube form a young age and engaging in substantial amounts of usage, so we need to study the early childhood age group, which is younger than teenagers [1, 2, 21].

For these reasons, this study initially aims to investigate the current state of YouTube usage during early childhood, examining the association between early childhood temperament—a dispositional factor that may influence YouTube use patterns. Additionally, the research will explore the relationships between usage patterns including the age at first YouTube use, usage duration, usage frequency, and the associated emotional and behavioral problems. The findings will provide foundational data for future studies on YouTube, elucidating its unique characteristics by highlighting both similarities and differences with smartphones and social media. This will aid in understanding the distinctiveness of YouTube.

## Methods

### Study design and participants

This study focused on children aged between 8 and 11. The Kids Cohort for Understanding of Internet Addiction Risk Factors in Early Childhood (K-CURE) study is the first long-term prospective cohort study in Korea aimed at understanding the long-term effects of media exposure on infants and toddlers’ developmental trajectory and developing preventive and control measures. Every year this study tracks the developmental trajectory of 2- to 5-year-old infants and toddlers who participated in the first year. A measure of YouTube usage was added to the study in 2018 and was studied from Waves 4 to 7. This study was conducted as part of the K-CURE study and utilized data from Wave 4 (2018) to Wave 7 (2021).

In 2018 (Wave 4), the parent-reported Junior Temperament and Character Inventory (JTCI) was filled by the parents of 229 children. In 2021 (Wave 7), we measured children’s YouTube usage patterns by inviting caregivers (mostly mothers) to participate in a self-administered survey on children’s YouTube usage and their and their children’s mental health. The caregivers voluntarily visited community centers for children’s mental health in Suwon, Sungnam, or Goyang, all of which are major cities in the most populous provinces of Korea. Children’s emotional/behavioral problems were also measured in 2021 using the Korean version of the Child Behavior Checklist (K-CBCL). Children with serious developmental disabilities, such as autism or intellectual disabilities, were excluded from the study. After excluding 34 children who did not engage with YouTube, 195 children were included in the final sample.

## Measures

### Early childhood temperament

We used the JTCI to measure early childhood temperament of 5- to 8-year-old children in 2018. The JTCI is a version of the Temperament and Character Inventory (TCI) designed specifically for children and adolescents [22]. The TCI is questionnaire that measures temperament and character dimensions based on Cloninger’s biopsychological model [16]. The inventory measures 4 dimensions of temperament—novelty seeking, harm avoidance, reward dependence, and persistence—all of which tend to be relatively stable throughout one’s life. The JTCI has been validated in the Korean population. The Cronbach’s alpha of the 4 dimensions of the Korean version of the JTCI ranged from 0.48 to 0.80 [23].

### Age at first YouTube use and Subsequent YouTube usage patterns

We measured children’s YouTube usage in 2021 (Wave 7) using a questionnaire designed for 8- to 11-year-old children. The caregivers completed the questionnaire. Firstly, we asked what contents on YouTube their children mainly watch. Then, we asked the children’s age at first YouTube use. We provided the following options as the answer: under 4 years old, 4–5 years old, 6–7 years old, 8–9 years old, 10 or over 10 years old, and unsure. We also collected data on children’s subsequent YouTube usage patterns, which were measured based on usage frequency and duration. Usage frequency was measured in days per week by adding the number of days the child used YouTube on weekdays and weekends. We collected data on the average time spent using YouTube daily on weekdays and weekends and calculated YouTube usage duration in minutes/day in the following manner: (average daily usage on weekdays × days of use during the weekdays + average daily usage on weekends × days of use over the weekend) / 7.

### Emotional and behavioral problems

The Child Behavior Checklist (CBCL) is a standardized behavioral assessment scale completed by primary caregivers to evaluate social adjustment and emotional behavior problems in children [24]. CBCL is widely used worldwide, and the Korean version of the child Behavior Checklist (K-CBCL) has been standardized [25]. The K-CBCL includes 8 problem behavioral syndrome scales. Each item is rated on a 3-point scale from 0 to 2, and the scores are converted to T-scores, with higher scores indicating greater levels of problem behavior. The internal consistency Cronbach's alpha for the K-CBCL ranged from 0.62 to 0.95 [25].

### Statistical analysis

We evaluated descriptive statistics using the Statistical Package for the Social Sciences (SPSS), version 25.0, and inferential statistics using R version 4.3.0. First, we performed a frequency analysis of the participants’ demographic data and YouTube usage. Second, we conducted a correlation analysis to determine the relationships among the scores for temperament dimensions, the age at first YouTube use, YouTube usage patterns, and the total score on the K-CBCL. Third, we conducted a multiple linear regression analysis to examine the relationship between early childhood temperament and subsequent YouTube usage patterns. Finally, we performed a multiple linear regression analysis to determine whether early childhood temperament and the age at first YouTube use, subsequent YouTube usage patterns can predict emotional/behavioral problems after controlling for factors, such as sex, age, monthly household income, and parent’s employment status. A p-value less than 0.05 was considered statistically significant.

## Results

### Demographic characteristics and YouTube usage

Table 1 presents descriptive characteristics and YouTube usage. The sample was nearly sex-balanced with 50.8% males and 49.2% females. On average, the participants were aged approximately 117 ± 9.8 months. In the case of most participants, the monthly household income was USD 3000–4500 and one parent was employed. For reference, the average monthly income of Korean families in 2021 was approximately 4,081,600 won, with a median of 4,965,800 won, and a standard deviation of 46,300 won. The income distribution was as follows: 12.38% earned less than 2 million won, 27.37% earned between 2 to 4 million won, 29.80% earned between 4 to 6 million won, and 30.45% earned over 6 million won. Our analysis reveals that the proportion of families from the lowest income bracket in our study sample is relatively small, which may influence the interpretation of our findings. Among content categories, gaming was the most preferred by children, followed by DIY (Do it yourself), which was the second most preferred, and film & animation, which was the third most preferred. 21.0% of children started using YouTube before age 4, with the most common onset age being 8–9 years (30.3%). The average frequency and duration of using YouTube was 4.8 days/week and 68.5 min/day, respectively**.**

**Table 1:** Demographic characteristics and YouTube usage (*n* = 195)

Variables	n or mean	% or SD
Sex, female	96	49.2%
Age, month (SD)	117	9.8
Monthly household income		
~ $1500 (2million KRW)	6	3.1%
~ $3000 (4million KRW)	37	19.0%
~ $4500 (6million KRW)	77	39.5%
greater than $4500	75	38.4%
Parent's employment status		
One parent work	103	52.8%
Both parents work	92	47.2%
Mainly watching contents		
Gaming	58	29.7%
DIY (Do it yourself)	57	29.2%
Film & Animation	27	13.8%
Education	16	8.2%
Music	10	5.1%
Video Blogs	10	5.1%
Others	17	8.7%
Age at first YouTube use		
before the age of 4	41	21.0%
4 ~ 5 years old	28	14.4%
6 ~ 7 years old	50	25.6%
8 ~ 9 years old	59	30.3%
10 years old	11	5.6%
missing value	6	3.1%
Average usage frequency (days/week)	4.8	2.3
Average usage duration (min/day)	68.5	48.4

### Correlation analysis among temperament, YouTube usage and total K-CBCL score

Table 2 presents the results of the correlations between early childhood temperament, subsequent YouTube usage, and emotional/behavioral problems represented by the K-CBCL scores. Persistence was negatively correlated with subsequent YouTube usage duration (*r* = -0.241). Age at first YouTube use was negatively correlated with usage frequency (*r* = -0.159) and total score on the K-CBCL (*r* = -0.172). Further, usage frequency was positively correlated with total score on the K-CBCL (*r* = 0.160).

**Table 2:**
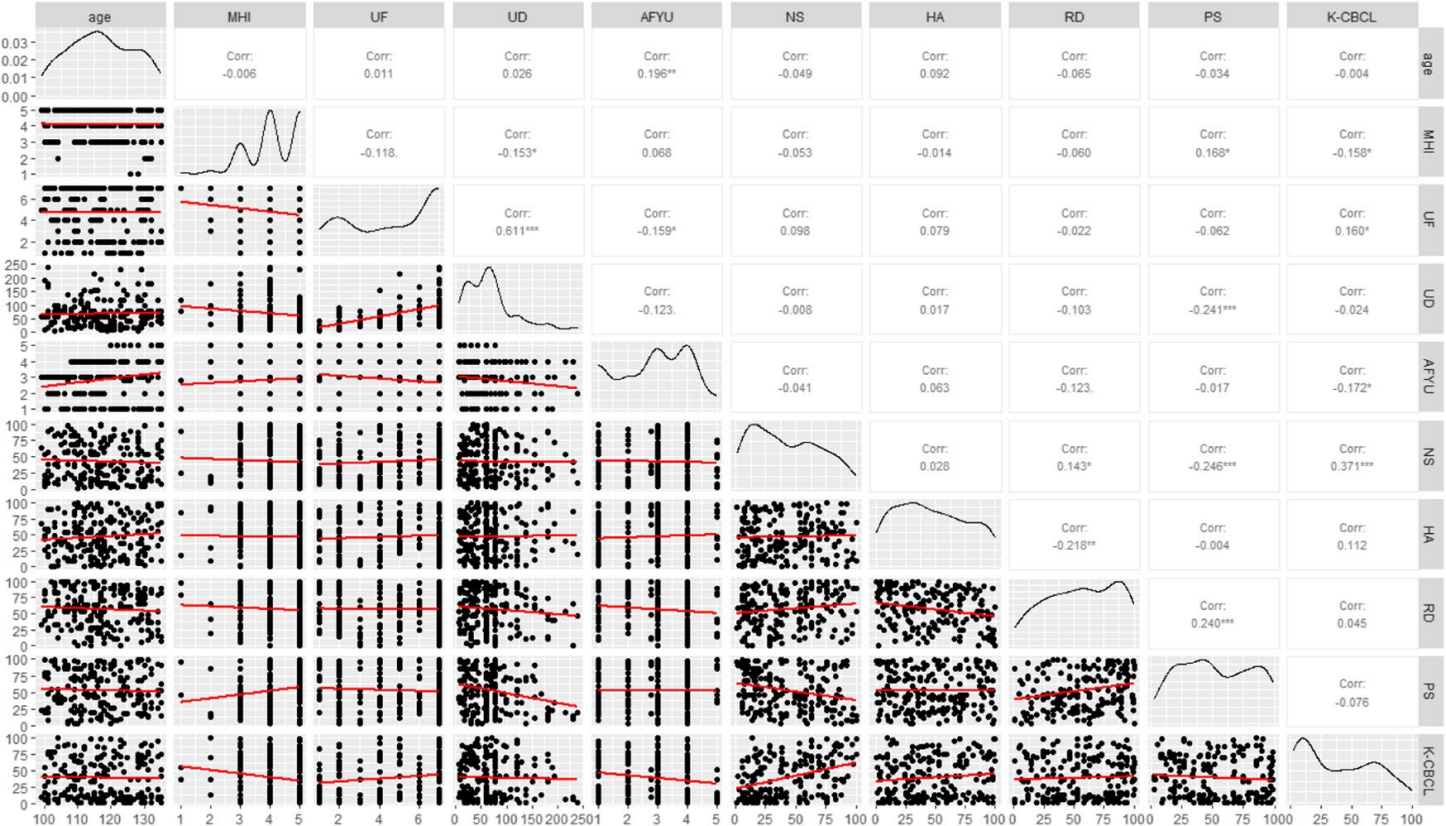
Correlation analysis among temperament, YouTube usage and total K-CBCL score

*P < 0.05, ** *P* < 0.01, *** *P* < 0.001

*Abbreviations*:* MHI *Monthly Household Income, *UF *Usage Frequency, *UD *Usage duration, *AFYU *Age at first YouTube use, *NS *Novelty Seeking, *HA *Harm Avoidance, *RD *Reward Dependency, *PS *Persistence, *K-CBCL *Korean Version of The Child Behavior Checklist

### Multiple linear regression between temperament and subsequent YouTube usage patterns

Table 3 presents the results of association between early childhood temperament and subsequent YouTube usage duration. Persistence was found to be significantly associated with subsequent YouTube usage duration (Standardized coefficient, β = -0.258, *P* < 0.01). Table 4 presents the results of association between temperament and subsequent YouTube usage frequency. However, this model was not statistically significant.

**Table 3:** Multiple linear regression analysis results of YouTube usage duration using temperament subscale

Model Fit Measures										
							Overall Model Test			
Model	R	R²	Adjusted R²	AIC	BIC	RMSE	F	df1	df2	p
1	0.349	0.122	0.079	2062	2098	45.3	2.84	9	185	0.004
Coefficients		Stand. β		β		Std. Error		t		P
(Intercept)				125.863		45.346		2.776		0.006**
NS		-0.086		-0.150		0.128		-1.168		0.244
HA		0.001		0.002		0.117		0.016		0.987
RD		-0.060		-0.101		0.128		-0.791		0.430
PS		-0.258		-0.434		0.130		-3.349		< .001**
Age at first YouTube use		-0.152		-6.008		2.817		-2.133		0.034*
age(month)		0.035		0.171		0.349		0.491		0.624
sex		-0.083		-3.997		6.829		-0.585		0.559
Monthly household income		-0.140		-7.899		4.048		-1.951		0.053
Parent's employment status		0.344		16.643		7.185		2.316		0.022*

*P < 0.05, ** *P* < 0.01

The model was controlled for age at first YouTube use, age, sex, monthly household income, and parent’s employment status

*Abbreviations: NS *Novelty Seeking, *HA *Harm Avoidance, *RD* Reward Dependency, *PS* Persistence

**Table 4:** Multiple linear regression analysis results of YouTube usage frequency using temperament subscale

Model Fit Measures										
							Overall Model Test			
Model	R	R²	Adjusted R²	AIC	BIC	RMSE	F	df1	df2	p
1	0.26	0.068	0.022	877	913	2.17	1.49	9	185	0.154
Coefficients		Stand. β		β		Std. Error		t		P
(Intercept)				5.655		2.172		2.604		0.010*
NS		0.073		0.006		0.006		0.963		0.337
HA		0.068		0.005		0.006		0.922		0.358
RD		-0.039		-0.003		0.006		-0.505		0.614
PS		-0.038		-0.003		0.006		-0.472		0.637
Age at first YouTube use		-0.175		-0.323		0.135		-2.391		0.018*
age(month)		0.036		0.008		0.017		0.499		0.618
sex		-0.123		-0.276		0.327		-0.845		0.399
Monthly household income		-0.117		-0.306		0.194		-1.577		0.116
Parent's employment status		0.231		0.520		0.344		1.510		0.133

*P < 0.05, ** *P* < 0.01

The model was controlled for age at first YouTube use, age, sex, monthly household income, and parent’s employment status

*Abbreviations: NS *Novelty Seeking, *HA *Harm Avoidance, *RD *Reward Dependency, *PS *Persistence

### Multiple linear regression among temperament, YouTube usage and total K-CBCL score

Finally, we examined the association among temperament, age at first YouTube use, usage patterns, and emotional/behavioral problems. The findings of this analysis are detailed in Table 5. The age at first YouTube use was negatively associated with the total score on the K-CBCL (Standardized coefficient, β = -0.155, *P* < 0.05). YouTube usage frequency was positively associated (Standardized coefficient, β = 0.187, *P* < 0.05) and usage duration was negatively associated with the total score on the K-CBCL (Standardized coefficient, β = -0.176, *P* < 0.05). The regression model accounted for 17.2% of the variance in the outcome.

**Table 5:** Multiple linear regression analysis results for total K-CBCL scores using YouTube usage and temperament

Model Fit Measures										
							Overall Model Test			
Model	R	R²	Adjusted R²	AIC	BIC	RMSE	F	df1	df2	p
1	0.468	0.219	0.172	1859	1901	26.6	4.56	11	183	<.001
Coefficients		Stand. β		β		Std. Error		t		P
(Intercept)				33.653		24.427		1.227		0.221
Age at first YouTube use		-0.155		-3.815		1.692		-2.254		0.025*
Usage Frequency		0.187		2.505		1.140		2.197		0.029*
Usage Duration		-0.176		-0.110		0.055		-2.006		0.046*
NS		0.344		0.372		0.077		4.846		< .001**
HA		0.091		0.093		0.069		1.341		0.182
RD		-0.025		-0.026		0.076		-0.348		0.728
PS		0.010		0.010		0.080		0.125		0.901
Age (month)		0.036		0.110		0.206		0.534		0.594
Sex		-0.042		-1.277		4.041		-0.316		0.752
Monthly household income		-0.129		-4.523		2.417		-1.871		0.063
Parent's employment status		-0.043		-1.295		4.305		-0.301		0.764

*P < 0.05, ** *P* < 0.01

The model was controlled for age, sex, monthly household income, and parent’s employment status

*Abbreviations: NS *Novelty Seeking, *HA *Harm Avoidance, *RD *Reward Dependency, *PS *Persistence, *K-CBCL *Korean Version of The Child Behavior Checklist

## Discussion

This study was conducted to first assess the current state of YouTube usage among children and adolescents, then examined the relationship between early childhood temperament as potential influencing factor on subsequent usage patterns, and finally investigated the association of these usage patterns with emotional/behavioral problems. Firstly, we found that 21.0% of children started using YouTube before age 4, with the most common onset age being 8–9 years and use it 4.8 days per week for 68.5 min per day on average. South Korean studies involving 6-year-olds and younger children have found that these children tend to use YouTube for the first time before the age of 2 or 3, and the younger the child, the younger the age at first YouTube use [26, 27]. In this study, the most common age of first YouTube use was 8–9 years, a higher starting age than that found in these previous Korean studies. This difference may be because previous Korean studies focused on 6-year-olds and younger children, but we focused on older children, and the younger the child, the younger the age at first use of YouTube. And a South Korean study involving 238 children of the similar age as our participants found that many children use YouTube almost daily, with most spending, on average, more than an hour daily on YouTube [28].

Our second finding was that early childhood persistence is negatively correlated with subsequent YouTube usage duration. Persistence refers to one’s propensity to persevere in the absence of rewards. Highly persistent individuals are considered hardworking and stable, whereas less persistent ones are considered lazy and unstable [29]. Rothbart defined temperament as individual differences in innate biological reactivity and self-regulation. She emphasized that the ability to self-regulate is also based on temperament and proposed a new dimension of temperament called effortful control [30]. On the other hand, studies on smartphone usage have found self-control to be a determinant of excessive or problematic smartphone usage [31, 32]. The temperament dimension underlying this self-control is effortful control, and a similar concept among Cloninger’s temperament dimensions might be persistence [29]. Previous studies on temperament and problematic smartphone usage have shown high levels of novelty seeking, harm avoidance, reward dependence, and low levels of persistence are associated with problematic smartphone usage. Among these, novelty seeking and harm avoidance are the main outcomes [33, 34]. However, we did not obtain similar results. Previous studies have generally examined excessive or problematic smartphone usage as the dependent variable, whereas our study focused on usage patterns. Moreover, previous studies have used questionnaires to examine temperament at the time of smartphone usage and primarily targeted late teenagers or college-aged adults. The fact that this is the first study to examine the relationship between early childhood temperament and children's subsequent usage patterns on YouTube may have contributed to some of the unique aspects of the results.

Our findings also may be related to the importance of self-control in early childhood. While gaming, the Internet, and smartphone use involve a more active search for content, YouTube is slightly different because users consume videos that pop up on the screen or are recommended by YouTube’s algorithm. Since algorithms can boost video consumption even more, persistence, which is related to self-control, may be crucial. However, the model’s explanatory power is relatively low (adjusted R-squared value of 0.079). Although the results show that temperament does not have a high explanatory power in explaining children’s YouTube usage patterns, this does not mean that the results of this study are meaningless, as the usage patterns of YouTube are influenced by many factors including temperament. The fact that this study is the first to examine the association between temperament as a dispositional variable and subsequent usage patterns on YouTube is significant and will provide basic knowledge about children's usage patterns in the future. However, it is not possible to conclusively determine the relationship between temperament and YouTube usage based on the results of this study alone. Therefore, it will be necessary to conduct further research by increasing the sample size or using other methodologies.

Our third finding was that the age at first YouTube use is negatively correlated with subsequent YouTube usage frequency, and these two variables significantly affects emotional/behavioral problems. Few studies have directly examined the association between age at first use and subsequent usage patterns even in smartphone research. In previous studies, researchers have divided participants into two groups: those engaging in excessive or problematic usage and those who do not. They examined the association between the age at first smartphone use and these groups. It has been predominantly observed that the younger the age at first smartphone use, the higher the likelihood of addiction [35, 36]. Infancy and early childhood are critical periods of growth and development. When there is considerable plasticity [13], the impact of media exposure on physical and mental health can be even more pronounced and irreversible. Previous studies have shown that early exposure to digital media and smartphones is associated with decreased cognitive function, language delays, and emotional/behavioral problems [11, 12, 37]. Children can become engrossed in the enjoyment they derive, leading to excessive use of smart devices. Young children without proper cognitive judgment may also unintentionally be exposed to violent and inappropriate visual content through what they watch or due to YouTube’s algorithm [38], potentially increasing the likelihood of emotional/behavioral problems later on.

Also, higher usage frequency was significantly associated with increased emotional/behavioral problems. This finding aligns with that of previous studies because they have found that excessive smartphone usage induces negative emotions, such as depression and anxiety, and hampers overall quality of life [5, 6]. Early studies on smartphone usage focused on the amount of time spent on smartphones. However, topics have gradually expanded to include social media, and the research has become more specific about usage patterns, examining how different devices are used, what platforms are engaged with, how long and how frequently they are used based on user motivation. Recent research suggests that the frequency of smartphone usage may have a greater impact on smartphone dependence than the amount of time spent on smartphones [39–41]. Furthermore, recent studies have obtained contradictory results or varying effect sizes concerning the relationship between smartphone usage duration and emotional/behavioral problems, mental health [42–44]. In these studies, frequent usage is said to impact self-regulation, potentially leading to more addictive behavior. Additionally, previous smartphone studies have primarily targeted late adolescents or early adults due to high penetration and usage rates. However, topics such as the link between excessive use and greater emotional/behavioral problems, fundamentally explored in smartphone research, have not scarcely investigated in the context of YouTube. Although our research may revisit themes commonly addressed in smartphone studies, it would be meaningful to conduct such a study specifically for YouTube, given the lack of existing research on these topics. Due to the ubiquitous nature of smartphones, children are using devices more freely and frequently. Nowadays, children tend to use devices and content for much shorter but more frequent periods, showing a preference for so-called “short-form videos” lasting less than 60 s [45, 46]. This phenomenon leads to decreased patience and self-control as children use devices and content more frequently to satisfy their desires, and frequent use can lead to reduced concentration and increased distractibility, thereby causing emotional/behavioral problems [39–41]. While our study's findings are consistent with recent smartphone and social media research, they are particularly significant because they focus on young children and YouTube. This suggests that the importance of qualitative factors such as usage motivation, content, and specific usage patterns is increasingly outweighing the simple quantitative concept of usage duration.

Our final finding was that YouTube usage duration is negatively associated with emotional/behavioral problems. This means that the longer children use YouTube, the fewer emotional/behavioral problems. However, correlation analysis did not show a correlation between YouTube usage duration and total score on the K-CBCL. Notably, this result differs from that of previous studies and defies conventional expectations [5, 39]. A detailed examination of the data used in this study revealed that the group with very extensive YouTube usage durations displayed significantly lower scores on the K-CBCL compared to other groups. The extreme nature of this data may have influenced a negative association between usage duration and K-CBCL scores. Specially, given the scarcity of research directly examining the relationship between YouTube usage duration and children's emotional/behavioral problems, this study challenges the conventional findings that longer screen time is associated with high emotional/behavioral outcomes. However, the generalized concept of 'screen time' often fails to account for the diverse activities performed on digital devices. In our contemporary digital era, screens are pivotal for communication, information gathering, professional activities, and educational processes. Particularly, YouTube serves not merely as a media consumption outlet but as a multifaceted platform, suggesting that extended usage durations might have varied implications. During the final years of this study, notably 2020 and 2021, the COVID-19 pandemic led to widespread school closures in Korea, prompting a shift to remote learning modalities where YouTube was frequently utilized as an educational substitute. This adaptation likely influenced the dynamics of screen engagement. Empirical evidence from the United States corroborates the educational utility of YouTube, where it is increasingly integrated into teaching strategies [47, 48]. Furthermore, recent evaluations of YouTube's content, such as those by Neumann and Herodotou [49], have developed criteria to assess the suitability of videos for children, considering age appropriateness, content quality, design features, and educational objectives. These studies provide a curated list of recommended content, reinforcing the platform's potential in supporting child development. As such, YouTube, unlike other platforms and social media, plays a multifaceted role in expanding experiences and educating, so it is possible that the very extensive usage duration group is using YouTube in a variety of positive roles beyond just entertainment, social networking. The extreme nature of the data, the impact of COVID-19, and the evolving role of YouTube may have influenced the results of this study. Alternatively, the findings of this study that high usage frequency of use and short usage duration are associated with high emotional/behavioral problems may suggest a YouTube-specific difference. Given the lack of research on the association between YouTube use and emotional/behavioral problems, it is not possible to draw conclusions about the relationship between usage duration and emotional/behavioral problems based on this study alone, and further research should be conducted to further explore the relationship.

Initially, smartphone research primarily concentrated on screen time in terms of device usage duration and its correlation with mental health. Over time, however, the focus has shifted from merely quantifying usage duration to a more nuanced understanding that includes the type of devices used, the motivations behind their use, the platforms engaged with, and the specific usage patterns. Furthermore, as the use of social networking services has increased, research has expanded to encompass social media studies. Unlike the passive receipt of information that characterized earlier media consumers, modern users actively seek out necessary information, selectively engaging with specific media and services. Despite its status as one of the most utilized platforms globally, research on YouTube remains markedly scarce. This gap exists not only for topics that have been extensively explored in traditional smartphone studies but also for issues unique to or beyond YouTube. YouTube shares similarities with social media platforms like Facebook, Instagram, and Twitter, yet it also possesses distinct characteristics. Unlike other social media that primarily facilitate social networking, YouTube serves as a versatile platform used from early childhood for various purposes, including play participation and learning. Furthermore, YouTube centers around video content and fosters bi-directional interactivity with features like Likes and Subscribe, positioning itself as a critical broadcasting medium potentially replacing traditional media [50]. As a result, parents with young children increasingly turn to YouTube to meet a variety of childrearing needs, including enriching their children's experiences, facilitating learning, and even reducing problematic behaviors. Consequently, screen viewing via YouTube has become a routine activity for infants and toddlers [21].

While YouTube's use of the Internet and smartphones may be similar to other smartphone studies, the platforms used by smartphones are different, and the results will not necessarily be the same as those of other smartphone and social media studies. Our study's results confirm this variation. In previous smartphone and social media studies, important temperaments were mainly novelty seeking and harm avoidance, but in this study, persistence was found to be a significant temperament. In addition, within the context of the study results, it is interpreted that in terms of YouTube usage, usage frequency is more meaningful than usage duration. On YouTube, the recommendation algorithm automatically displays suggested videos, increasing consumption by providing content based on previously watched videos, which are likely to align with the user's preferences. This process naturally leads to an increase in the amount of content consumed. Therefore, rather than novelty seeking, which involves pursuing new and diverse content, the trait of persistence—referring to the ability to regulate and control the consumption of related videos—may be a more significant characteristic to consider. Furthermore, recent research into excessive or problematic smartphone use has shown that usage frequency is yielding more significant results than usage duration [39–41]. This may be due to smartphones' exceptional accessibility, which allows children to instantly satisfy their desire to use the device, leading to short but frequent sessions that can enhance addictive behaviors and impair self-regulation [39–41]. This is particularly significant as children and adolescents are in a developmental stage where self-regulation is not yet fully matured [51]. In summary, the findings of this study may reflect the importance of self-regulation in children and adolescents. However, considering that most social media platforms and digital platforms today are equipped with algorithms for recommendations and autoplay, and given the prevalent usage pattern among youth of using these platforms frequently but for short durations, the results of this study may not be limited to YouTube alone. Rather, they could indicate significant traits or attitudes necessary for children and adolescents when engaging with platforms in the current digital era. This study is the first to focus on YouTube, a previously under-researched area, using the JTCI to investigate the relationship between early childhood temperament, subsequent YouTube usage patterns, and emotional/behavioral problems. Furthermore, this study serves to re-examine topics previously explored in the context of smartphones and social media within the realm of YouTube. The results provide valuable insights into the similarities and differences between smartphones and YouTube, underscoring the significance of understanding these distinctions.

Despite its significance, this study has some limitations. First, all questionnaires in this study were completed by caregivers. Second, we did not consider various other factors that could influence results. Particularly, this study did not consider the content consumed, which could be a critical qualitative factor, nor did it take into account parental monitoring, a variable previously identified as significant in other studies [52, 53]. Moreover, the YouTube usage frequency observed in this research may be related to household smartphone usage rules, taking into account the age of the participants. Finally, this study was conducted in South Korea, and we must consider the fact that smartphone penetration is high in South Korea. However, since our results align with those of previous studies involving children of similar age groups, it is difficult to argue that our data are not objective. Nonetheless, follow-up studies should consider utilizing objective data, such as smartphone logs and YouTube viewing records. Future studies must also conduct quantitative and qualitative analyses on various aspects, including video quality, and the influence of algorithms, parental monitoring, the type of content consumed by children and adolescents. Despite these limitations, this study is significant as it focuses on YouTube and examines the relationships among children's temperament, subsequent usage patterns, and emotional/behavioral problems. It provides foundational information that will be valuable for future research on YouTube.

## Conclusions

We found that early childhood persistence is negatively associated with subsequent YouTube usage duration in this study. Additionally, age at first YouTube use is negatively correlated with usage frequency, and these two variables significantly affect emotional/behavioral problems. Moreover, children’s YouTube usage was much higher than expected. Some findings related to smartphone usage might apply to YouTube, but with some differences. Our results can be utilized in developing guidelines for appropriate YouTube use among children and adolescents. Furthermore, future research is necessary on the complex interplay between YouTube usage, mental health, and various influencing factors in children and adolescents.

## Data Availability

The datasets used and/or analyzed during the current study ar available from the corresponding author on reasonable request.

## Abbreviations

K-CURE: Kids Cohort for Understanding of Internet Addiction Risk Factors in Early Childhood
TCI: Temperament and Character Inventory
JTCI: Junior Temperament and Character Inventory
NS: Novelty seeking
HA: Harm avoidance
RD: Reward dependency
PS: Persistence
K-CBCL: Korean version of the Child Behavior Checklist
DIY: Do It Yourself
